# Human Complex Trait Genetics: Lifting the Lid of the Genomics Toolbox - from Pathways to Prediction

**DOI:** 10.2174/138920212800543101

**Published:** 2012-05

**Authors:** Suzanne J. Rowe, Albert Tenesa

**Affiliations:** 1 The Roslin Institute, The University of Edinburgh, Easter Bush Campus, Midlothian, EH25 9RG, Scotland, UK; 2 Institute of Genetics and Molecular Medicine, 4th Floor, MRC Human Genetics Unit, Western General Hospital, Crewe Road South, Edinburgh, EH4 2XU, UK

**Keywords:** Association complex human genetics genome-wide genomics GWAS prediction.

## Abstract

During the initial stages of the genome revolution human genetics was hugely successful in discovering the underlying genes for monogenic diseases. Over 3,000 monogenic diseases have been discovered with simple patterns of inheritance. The unravelling and identification of the genetic variants underlying complex or multifactorial traits, however, is proving much more elusive. There have been over 1,000 significant variants found for many quantitative and binary traits yet they explain very little of the estimated genetic variance or heritability evident from family analysis. There are many hypotheses as to why this might be the case. This apparent lack of information is holding back the clinical application of genetics and shedding doubt on whether more of the same will reveal where the remainder of the variation lies. Here we explore the current state of play, the types of variants we can detect and how they are currently exploited. Finally we look at the future challenges we must face to persuade the human genome to yield its secrets.

## INTRODUCTION

Complex traits are determined by the interplay of multiple genetic and environmental factors. Understanding the interaction of nature and nurture in the development of common human disease and continuous traits is the main interest of complex trait human geneticists. Unlike monogenic traits, complex trait variation is not entirely explained by one or a small number of genes but results from a complex mixture of inherited and environmental factors. The proportion of phenotypic variation explained by inherited genetic factors is known as the heritability [1], and this measures the degree of resemblance among relatives [2]. Twin and other family-based studies have shown that genetic variation explains a large proportion of the phenotypic variation observed in humans. Heritability estimates vary widely among traits, 40% of variation of most complex traits can be explained by inherited factors increasing to over 70% for some diseases such as schizophrenia [3]. 

**Fig. (1):**
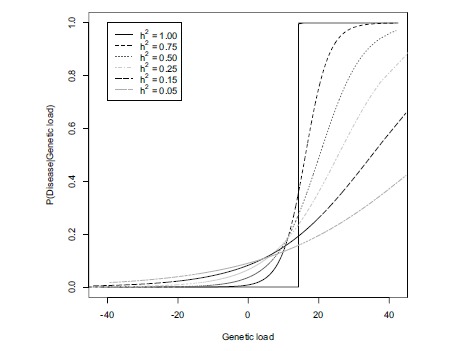
Penetrance, the probability of disease given genetic load, for diseases with the same prevalence (0.1) and varying heritability (h^2^).

Genetic variants, however, often predispose us to, rather than cause disease, and even more complications arise as they predispose us in combination with other variants and environmental influences. Penetrance is usually defined as the probability of disease given the genotype of a person, however for complex disease, it makes more sense to define penetrance as a function of total genetic load where each genetic variant is weighted according to the risk it confers (Fig. **1**). Furthermore phenotypic variation comes in many layers of biological complexity at the cellular, tissue and organism level that are likely to be involved in the pathogenesis of disease. Since disease is in itself complex, it is useful to study, and in many cases clinically treat, some intermediate disease endpoints that can be measured before the onset of the disease. For instance, high blood pressure and LDL cholesterol are treatable intermediate disease endpoints for myocardial infarction or stroke. There are multiple sources of genetic variation linked to phenotypic variation, which include multiple layers of biological complexity that lead to disease. Understanding how these are controlled by DNA alterations that are transmitted from parents to their children is one of the fundamental challenges of modern biology. 

## COMPLEX TRAIT MAPPING

The use of statistical methods to pinpoint the regions of the genome that harbour the DNA changes and genes that control complex traits is known as mapping. Early linkage studies exploited repetition in the genome by using cutting enzymes to identify markers that might be co-inherited with causal variants thus providing clues as to the locations in the genome affecting traits of interest. The highly polymorphic and co-dominant microsatellite markers were technically easier to work with and quickly replaced restriction fragment length polymorphisms (RFLP) in linkage studies aiming to map complex trait loci (CTL).

There are two broad approaches to CTL mapping in human family-based linkage studies. Either sampled pairs of sibs, or large sets of relatives from extended or nuclear families are analysed. The methodology relies on using marker information to measure how related individuals are at the putative CTL. This measure of identity is termed identity by descent (IBD) where individuals share copies of a gene inherited from a common ancestor. Phenotypic similarity between two individuals is modelled as a function of their relatedness at each test position. The most widely used non-parametric method for linkage analysis of quantitative traits is sib pair analysis based on the regression method of Haseman and Elston [4]. The squared difference between the trait values for a pair of relatives is regressed against the proportion of marker alleles IBD. A negative co-efficient reflects a tendency for individuals to be more similar with respect to the trait as they share a greater proportion of the alleles IBD thus implying linkage between trait and marker. Different allelic and causal variant combinations may be seen across families, therefore, data is analysed within families and information combined across multiple families. An advantage of microsatellite markers is that they are highly polymorphic and with phased data, models can easily be extended to incorporate interactions such as dominance, epistasis and imprinting. Methods for tracking the transmission of alleles from parents dependent on affected status **– **and that exploit linkage disequilibrium (LD) within families and at the population level **– **include the transmission disequilibrium test (TDT) [5] and the family based association test (FBAT) [6] These methods have been further adapted for quantitative traits(QTDT) [7]. Family based linkage studies provided a powerful approach for identifying rare variants of large effect segregating within individual families and proved very successful for simple or monogenic traits. For complex traits, however, the approach has yielded little fruit and few of the reported findings have been successfully replicated. Lack of reproducible associations raised the question, as it does now with GWAS, of whether the approach had reached its limits. In a seminal paper, Risch and Merikangas [8] argued that modest gene effects were likely to explain the lack of reproducible linkage results and that a new approach would be required to overcome the next hurdle of human complex traits genetics. 

The common disease/common variant (CD/CV) [9] hypothesis states that common complex diseases are underpinned by a large number of common variants of small effect segregating in the population, rather than a small number of rare variants of large effect. In order to fine map the CTL found in linkage studies and in particular to look for variants assumed to be common in the population, association mapping was born. The aim is to look for over representation of marker genotypes associated at a population level with a particular disease or phenotype. This necessitates a very dense marker map with adjacent markers in high LD in order to track ancestral haplotypes after many generations of recombination. It is estimated that there is at least 1 single nucleotide polymorphism (SNP) every 100-300 base pairs with greater than 1% minor allele frequency (MAF) [10] making them ideally suited as markers to tag causal variants. 

The use of association for fine mapping candidate regions from linkage studies quickly gave way to more general or ‘genome-wide’ association studies (GWAS). One of the greatest benefits of GWAS is that it is ‘agnostic’ or based on no prior assumptions. Generally a simple regression analysis is used to systematically test each biallelic SNP across the genome for association with a trait or disease. Many generations of recombination creates smaller regions of LD which with a dense-enough marker coverage provides a much higher resolution than linkage and the potential to tag common causal variants. Care must be taken to ensure that the associated loci are not spurious associations due to, for example, population substructure or admixture [11]. The use of hundreds of thousands of markers also necessitates very strict significance criteria making it difficult to detect all but the largest effects. 

To facilitate association mapping the HapMap project was developed, the second phase of which identified more than 3.1 million SNPs, from 270 individuals from 4 populations [10]. Phase 3 offered a further 1.6 million SNPs and expansion to a total of 11 populations and 1184 individuals [12]. The abundance of these SNPs means that most common SNPs are in high LD with neighbouring SNPs with an average minimum r^2^ squared value of between 0.9 and 0.96 depending on population providing excellent coverage of common variation across the genome. The reference panels of the Hapmap project are routinely used to statistically estimate (i.e. impute) genotypes within marker intervals of sparser data sets [13-16]. This aids the meta-analysis of multiple cohorts genotyped with different genotyping arrays.

## SUCCESS FROM GENOME-WIDE ASSOCIATION STUDIES

The initial maelstrom of GWAS began in 2005/2006. Fig. (**2**) shows that since 2006 the number of published SNPs exceeding genome-wide significance from GWAS has risen linearly. The National human genome Research Institute (NGHRi) database of published results contains 5118 entries to date affecting over 500 traits (http://www.genome.gov/gwastudies) [17]. Fig. (**3**) shows the top 30 diseases with the greatest number of entries. There have been more than 90 cancer susceptibility loci identified [18], over 180 loci for height [19], 39 for type 2 diabetes [20] and 71 for Crohn’s disease [21]. With commercial arrays it is now commonplace for published studies to involve analyses using over 500,000 SNPs.

Despite these successes much of the heritability or genetic variance estimated to exist remains unaccounted for [22-24] (Table **1**). Complex traits are often associated with high heritability yet there is mounting empirical evidence from GWAS results that there are few common variants of large effect. Fig. (**2**) shows the median odds ratio for the NHGRi database is only 1.1. Height is often used as an example as although 180 loci have been found above the genome wide significance level, these variants only explain around 12% of the genetic variance [19]. Studies rarely seem to find variants explaining more than 1% of sibling recurrence risk. There are of course exceptions such as large variants found for Crohn’s disease with an odds ratio of 3.99 [25] and activated partial thromboplastin time where three common variants explain 18% of the phenotypic variance [26]. It is increasingly likely, however, that most large common variants have now been discovered, therefore, the crucial question is how to capture the remaining variation - often dubbed the missing heritability or the dark matter of the genome. Furthermore, if we have already discovered the low hanging fruit, are there diminishing returns to be expected from further GWAS?

Although a proportion of the missing heritability may potentially be due to inflation of estimates of additive variance due to other non-linear sources of variation such as epistasis, epigenetics and gene by environment interaction [2,27], this is, in general, not supported by theoretical and empirical data [28]. Even if epistasis was widespread, its detection would be challenging due to the number of tests involved, lack of power and the difficulty of setting appropriate significance thresholds. These difficulties are reflected in the little evidence available from large genome-wide association studies to suggest interlocus interactions. It is highly probable that there are a large number of loci with effects too small to achieve significance by the stringent thresholds set. It is also possible that the remaining variants are rare and therefore poorly tagged by current arrays, which use common tag SNPs unlikely to be in sufficient LD with rarer variants. More powerful methodology can capture this hidden genetic variation by using all SNP information regardless of significance thus avoiding the problem of stringent significant thresholds set in GWAS. Yang *et al*. [22] used ~295K SNPs on ~4K individuals and explained 10-fold more variation of height than previously reported (i.e. the ~295K SNPs explained about 45% of variance). Furthermore if the incomplete LD caused by the differences between the distributions of minor allele frequency for SNPs and causal variants is accounted for, 80% of the variation of height can be explained in line with literature estimates of narrow sense heritability. The approach of Yang *et al*. [29] can be readily extended to partition the genetic variance for designated regions (often per chromosomes) by using a linear mixed model to fit all SNPs in that region simultaneously. This provides a mechanism for locating regions of importance containing markers or groups of markers which may not meet significant thresholds on an individual basis.

Single marker based association models can also be extended further to incorporate combinations of markers or haplotypes. If the distribution of CTL differs from that of markers, the utilisation of haplotype information could capture associations with rarer variants eluded by single SNP analysis because allele frequencies at the CTL and the ‘virtual’ marker created by the haplotype will be better matched [30,31]. Combinations of multiple SNPs or haplotypes could potentially capture greater proportions of genetic variation than single SNPs [32]. 

A further explanation for the lack of genetic variation captured by GWAS is that the allelic architecture underlying complex traits is not described accurately by the CD/CV hypothesis. The common disease rare variant hypothesis (CD/RV) states that there are many low frequency variants of large effect segregating in the population and that each phenotype is due to combined effect of a few of these variants [33-35]. Under mutation selection balance theory the expectation would be an inverse correlation between deleterious SNPs and minor allele frequency with a probable upper limit of 1% applying to deleterious alleles [36]. Recent evidence for the (CD/RV) theory includes a rare variant in MHY6 associated with sick sinus syndrome or slow heart rate [37]. The risk allele has a frequency in the Icelandic population of 0.38% but has an associated odds ratio of 12.53. Lifetime risk in the population is 6%, however for carriers of the risk allele is 50%. Interestingly common variants of the gene modulate cardiac conduction. 

The CD/RV theory also supports variation due to widespread allelic heterogeneity. There is increasing evidence to suggest that multiple independent signals within a locus exist for a number of complex traits. There are a large number of disease causing allelic variants in some known genes such as BRCA1 and MLH1. Haiman *et al*. [38] found multiple independent regions associated with prostate cancer within the 8q24 locus and Lango Allen *et al*. [19] found that out of 180 loci significant for height at least 19 loci had multiple independently associated variants. Overall, this suggests that previously discovered loci are strong candidates for harbouring further missing genetic variation. 

The underlying distribution of effects in complex diseases may have a huge impact on the application of information discovered to date. One of the greatest hopes of GWAS was that it could be used for the detection of disease related CTL. Furthermore by identifying the genes involved there was much hope for the prediction and prevention of disease alongside new potential drug targets or therapeutics. If we were able to accurately estimate the effects of sufficient loci to explain half of the known genetic variance then genomic profiles for most common diseases would achieve sufficient discriminative ability to be of clinical validity. Even if accurately estimated loci explained only one quarter of genetic variance, for rare diseases (i.e. low prevalence) the genomic profile would be a more useful predictor of risk than self-reported family history [39]. These profiles can be used from birth enabling susceptible individuals to avoid environmental exposure to risk, thereby reducing absolute risk of disease.

The development of risk predictors for complex diseases has been slower than anticipated due to the small number of loci identified by current GWAS. For complex traits individual variants rarely explain enough variation to be utilised as risk predictors, however profiles based on many of these variants could potentially be used [40-42]. There is a mounting body of evidence showing that whole genome prediction methods developed over decades to estimate livestock breeding values offer the opportunity to increase the accuracy of prediction of disease risk [43-45]. Challenges include how to select and estimate the predictors of the model that minimises the mean square error of prediction of the phenotype. The genetic architecture of the trait determines the best strategy for model selection and the accuracy of the prediction model. Issues for model selection and expected accuracy include determining whether models that fit a subset of the available SNPs will perform better than models that fit all available SNPs simultaneously, how sensitive both approaches are to the misspecification of the genetic architecture of the trait [46,47], or the best strategy to shrink the estimates of the effects to prevent over-fitting of the models [48,49]. Furthermore in populations with high LD many loci will be correlated with each other affecting model choice and assumptions about prior distribution. Some of these issues are reviewed by Daetwyler *et al. *who give deterministic formulae for assessing the accuracy of genomic prediction [50]. 

The area under the receiver operator characteristic (ROC) curve (or its equivalent C-index) can be used to assess the discriminative ability of a prediction model [51] and has been used to assess the performance of genetic predictors and genomic profiling [52,53]. This is a technique for visualising, organising and selecting classifiers based on their performance often employed by the medical decision making community for diagnostic testing [54]. The performance of a diagnostic classifier over a range of thresholds can be examined to identify the threshold at which the classifier is most accurate. 

For disease prediction, the ROC curve represents the trade off between true positive rate (sensitivity) and false positive rate (1-specificity) and the area under the receiver operator characteristic curve (AUC) the probability that for a randomly selected case and control, the case will be ranked higher by the prediction model than the control. This is equivalent to the Mann–Whitney–Wilcoxon test statistic [55]. ROC curves are a useful measure as they are not affected by the skewness of the data i.e. they are not affected by the proportion of cases and controls (other than sampling error) which might vary from one data set to the next [51]. Wray *et al*., [56] give parameter estimates for 17 common complex diseases. They show that it is theoretically possible for a genomic profile for complex disease to exceed the threshold of discriminative ability of 0.75 that could, arguably, be considered clinically useful. They also show an AUC of 0.75 can imply anything from 0.1 to 0.74 of the genetic variance explained thus care should be taken in the interpretation of this statistic without some knowledge of the parameters used.

To explore this further we simulated data for prostate, breast and colorectal cancer to investigate the effect of various parameters on the prediction of risk. Ten thousand cases and 10,000 controls were simulated under a liability threshold model. The distribution of allele frequencies was taken from a beta distribution and the additive genetic effect sizes from a normal distribution. The data was randomly assigned to two equal sized groups, a training and a validation set. Three scenarios were examined a) the use of prediction or AUC when effects of all loci are known (i.e. we used the simulated effects), b) the use of prediction when the effects of all loci are estimated, and c) prediction using the 15 most significant loci. Results showed that, in particular, for the prediction of prostate cancer the discriminative ability across all ages (0.85) was higher than for the over 65’s (0.79). This is counter intuitive for a disease primarily of late onset but can be explained by the fact that the prevalence across all ages is lower, therefore those that have genetic risk factors leading to clinical diagnosis are likely to be at the extremes of the distribution in the population making the probability of discriminating accurately higher. Accuracy of prediction is very dependent on the underlying genetic architecture of the trait. Fig. (**4**) shows the discriminative ability for breast cancer (BC), colorectal cancer (CRC) and prostate cancer (PC) cancers either across all ages or in the over 65s comparing models with 500 or 10,000 underlying additive loci. When 10,000 loci were simulated the discriminative ability when using the 15 most significant effects ranged from 0.53-0.57. Whilst these figures are low and do not appear to offer a hopeful prognosis for clinical utility, recent results for 32 loci associated with BMI [57] found that although AUC for prediction of obesity was only 0.57 this was still an increase from using family and environmental information alone which only yielded an accuracy of 0.51. When we simulated a prevalence of 0.004, a heritability of 0.42 and 500 loci underlying the trait, the discriminative ability for a model which estimated the effects of all loci was 0.93 which was the equivalent of using the known simulated values for the loci. Accuracy using the top 15 most significant estimated effects was 0.75. In general the discriminative ability of the prediction models using the estimates of genetic effects are as good as using the actual simulated values. The maximum AUC of 0.92, 0.87, and 0.91, are similar to estimates from Wray *et al*. [56] who estimated 0.90, 0.89 and 0.96 for Prostate. Breast, and Colon cancer respectively using a threshold liability model. 

Park *et al*. [58] used summary statistics from existing GWAS to calculate the expected distribution and the number of loci that exist within the range of SNP effects observed on a trait by trait basis. They estimate discoveries for future GWAS for given sample sizes by integrating power over the number of unidentified loci that probably exist whilst accounting for the distribution of relative risk and allele frequency. Based on the assumption of a spectrum of low-penetrance common variants the predicted total number of loci within the range of effects currently detected by GWAS for height, Crohn’s disease and BPC (breast, prostate, colorectal) cancers are 201, 142 and 67 explaining 16.4, 20 and 17.1% of the genotypic variance respectively. They use these predictions to estimate the AUC for Crohn’s and the cancers. They predict that all 142 loci would give an AUC of 79.2% for Crohn’s in comparison to 72.8% from the 30 loci discovered to date. An AUC for breast cancer given the 5-10 loci that exist of 57% could be improved to 63.5% if all 67 loci were discovered. These results are based on modest estimates of heritability and MAF from published studies generally > 0.05 and do not reflect results from our simulation studies [59] or empirical data from Yang *et al*. [60].

A recent paper by Meuwissen *et al*., [44] takes genomic profiling a step further by exploring the prospect of prediction from whole genome sequencing data. They use simulation analyses to explore the accuracy of genomic prediction using sequence data which has the advantage of using all polymorphisms such as indels as well as SNPs. Furthermore the sequence data contains the causal variants. They conclude that if there are a finite number of causal variants a Bayesian approach is most successful, however should the distribution of effects follow an infinitesimal model with thousands of loci of small effects it is expected that BLUP methods will outperform the Bayesian analysis. Statistical methods for whole genome prediction are also reviewed by de los Campos *et al*. [43]. Ultimately, however, the success of prediction methods for complex diseases will be limited by the disease prevalence and the heritability. Even if the predictor explains 100% of the genetic variance, the maximum AUC is dependent on trait heritability ad prevalence. This is shown in Fig. (**2**) where even if the simulated values for all loci are used the AUC ranges from 0.87-0.93. Care must also be taken to note that genomic profiling infers genetic risk and not absolute risk. These profiles are a predictor of genotypic value rather than phenotype although they can be extended further to incorporate environmental risk factors such as smoking, diet or exercise. Furthermore, the discriminative ability of a model in a case-control study is different to discriminative ability at the population level and a model that is well calibrated for a case-control study may well yield a poor performance when screening the whole population. Strengths and weaknesses are further reviewed by Hand [55].

The advent of whole genome sequencing methods [61] provides the opportunity of increasing the information available for genetic mapping studies by the inclusion of all sources of genetic variation (SNPs, CNVs, indels, rearrangements, etc.) that may be causal and allows the unbiased screening of the genetic variation present in the sampled individuals. Of the 3200Mb of DNA in the human genome only 1.5% or ~50Mb is functional or coding DNA comprising approximately 20-25,000 genes. Large numbers of repetitive elements make up approximately 50% of the DNA. These include indels, copy number variants (CNVs), translocations, inversions, and chromosomal duplications. In a recent review of 75 cancer genes with germline mutations, 28 were reported to be mutated by genomic deletion or duplication [18]. 

The latest commercial arrays contain a combination of SNPs and structural variants [62]. CNVs have been associated with many traits [63] including starch digestion (AMY1) [64], HIV [62], Schizophrenia [65] and Crohn’s disease [66], although in European populations, most common CNVs are likely to have been tagged with SNPs. High heritability, high mutation rates and greater variation in African populations still provide compelling arguments for sequencing thousands more genomes and using CNVs to complement SNPs. 

Following the Hapmap project the thousand genomes project [10] http://www.1000genomes.org promises to deliver individual sequence variation by sequencing 1000 individuals. This will give insights into the variation in structural variation such as CNV’s, indels and deletions and into regulatory elements. The genomes of 2500 individuals from 25 populations around the world will be sequenced using next-generation sequencing technologies [61]. This international collaboration is set to produce an unprecedented public catalog of human genetic variation, including SNPs and structural variants, and their haplotype contexts to support genome-wide association studies and other medical research studies. The use of this valuable resource as a reference panel for imputation of cohorts with genome-wide association data may help to screen for rarer genetic variants. However, it is yet unclear whether rarer genetic variants will be accurately imputed using general reference panels. It may be more useful that each cohort generates its own reference panel by either sequencing or genotyping a subset of it for a dense SNP array. Even in the latter case, it is likely that the imputation quality of rare variants in low LD with common tagging SNP will be low, making re-sequencing of large numbers of samples necessary [67]. 

### Intermediate Phenotypes

It is becoming clear that SNP trait associations alone rarely lead to identifying the causal variant or the context in which the gene operates. This biological context is a necessary step for the generation of new biological hypotheses and the identification of drug targets for disease. There are many intermediate or endo-phenotypes which can be used to map complex variation. It is possible that these could be more heritable and represent a more comprehensive approach in the quest for the underlying causal variants of quantitative traits. 

The most widely studied intermediate phenotype is the study of expression analysis or the abundance of mRNA transcripts using microarrays or RNASeq to look at which genes are expressed at a given timepoint in a given tissue. Expression QTL may be categorised into cis (local) or trans (distant) effects. There appears to be a current bias towards large cis effects inferring regulatory processes are involved [68,69]. Associating patterns of gene expression with genotypic and phenotypic values facilitates insights into biological function. Genes which are differentially expressed can be used to infer regulatory networks and underlying pathways associated with traits or diseases [70,71].

Regulatory networks and discovered pathways in turn provide another dimension to GWAS and can themselves be used as intermediate phenotypes. Pathway enrichment analysis involves assigning SNPs to genes and subsequent pathways in order to find associations at the pathway level providing greater insight into biological function. Enrichment scores for known pathways can be obtained to investigate whether there is over representation of genes in any one pathway associated with phenotype [72-75]. The 180 loci discovered for height [19] are enriched for genes that are connected in biological pathways and underlie skeletal growth defects. Pathway analysis increasingly shows that pathways reputedly underlying common diseases are often common across diseases. It is difficult to ascertain whether this is due to annotation bias or whether there is genuine pleiotropy across the mechanisms underlying these diseases. It could be hypothesised some level of pleiotropy must exist given that there are approximately 21,000 genes and millions of traits. A recent study of coronary artery disease found that 5 out of 13 loci showed strong association with various other diseases or traits [76]. Interleukin receptor genes are linked with several common diseases such as Crohn’s, lupus, and rheumatoid arthritis implicating common underlying mechanisms or pathways [75]. The prediction of biological mechanisms seems at best tenuous with many potential biases not only from limitations of annotation but from setting of significance thresholds, assignment of variant to gene or pathway, algorithm or method used to ascertain enrichment scores, and fundamentally the type of biological data selected [77,78]. It is possible that in using a statistical approach we are much closer to the accurate prediction of phenotype using molecular data, which is almost a black box whole genome approach, than we are to gaining real insights into the specific underlying biological mechanisms which remain rather more elusive for most traits.

Finally, an important source of heritable variation not seen in coding sequence is epigenetic modification. This includes promoter methylation, histone tail modifications and altered expression of non-coding RNAs that associate with chromatin modifying complexes. These modifications contribute to gene regulation in normal development, particularly in foetal growth, to gene expression in tumorigenesis and have been shown to mediate the influence of environment on gene expression. Technologies are now sufficiently advanced to carry out methylation profiling. Gibbs *et al*., [79] find abundant QTL for DNA CpG methylation across the genome in brain tissue. Methylation studies are likely to play a wider role in the post genomic era. There are arguments, yet to be proven, for epigenetic variation as a driving force for development, evolutionary adaptation, and disease [80].

## CONCLUSIONS

Any GWAS is limited by depth of genomic coverage on commercial arrays. It remains to be seen whether results from current GWAS describe the full spectrum of existing genetic variation or merely what we have the ability to detect. It is highly probable that the latter is the case. The current status is that there are many variants detected but few explain more than 1% of trait variance and most genetic variance estimated by family studies is yet to be explained by allelic variation discovered from GWAS. Given extrapolation from current results it is likely that current studies are underpowered to detect all but the loci that explain the largest proportion of variance of complex traits. A common misconception is that rare variants of large effect will be easier to identify than common variants of small effect. Power depends on the proportion of variance explained by the SNP [31] and this is a function of the allele frequency and the effect. For instance, identified rare variants from GWAS (MAF 1-5%) have a mean OR ~3.74 [81] which, for disease prevalence ranging from 1-20% explain between 0.5-8% of the variance in the liability scale [82]. Identifying variants explaining this proportion of a quantitative trait variance at a genome-wide significance threshold of 5x10^-8^ with 80% power would require between 7900 and 460 samples, respectively. Finding a sufficient number of cases for some diseases in a single study or even at all may prove difficult and could be a limiting factor for future GWAS and whole-genome sequencing studies. As it is unlikely that the number of individuals needed to have enough power to detect these variants will come from a single study, the use of meta-analyses is and will be necessary to discover missing variants. Care must be taken that individuals are from the same population or that structure/admixture is properly identified and accounted for. It is important to note that whilst it is unlikely that increasing the SNP density of common variants will help to reveal new variants in European populations, there could still be much potential in populations of African descent where LD decays faster over distance and there is much more standing genetic variation.

New methods that exploit current GWA data are needed to tag rare variants which may be present in the population at lower minor allele frequencies than current SNP panels. Haplotype analysis may hold some promise if sufficiently large sample sizes are available. Poorly tagged structural and sequence variation may underlie some of the missing genetic variation and could explain allelic heterogeneity among common SNPs previously identified. 

Whole genome methods are increasingly likely to be used for the estimation of heritability and the clinical prediction of susceptibility to complex disease given the mounting body of evidence that traits are affected by hundreds if not thousands of variants. The main advantages of these methods are that all loci can be used simultaneously removing bias from setting stringent arbitrary thresholds to control for false positive rates. These whole genome methods assume additivity and there is currently little evidence to suggest otherwise, however, there may be gene-gene and gene-environment interactions yet to be uncovered. It is yet to be shown how accurate predictors will be within and across populations.

Whole genome sequencing and the thousand genomes project offer unprecedented opportunities to tag many more variants. The likely next steps before moving to large-scale whole-genome sequencing will be exome sequencing and whole-genome sequencing of a small number of samples from the population under study that will be used as the reference panel for imputation. It remains to be seen whether and under what circumstances this will provide any advantage to the use of publicly available reference sets such as the thousand genomes project. 

It is likely that the future of genomic mapping will involve incorporating methods such as re-sequencing and association analysis of all variants within a region of LD affecting a trait. To further elucidate the molecular and biological mechanisms involved it is likely that this will need to be followed by genomic analysis of gene expression and methylation in relevant human tissue and screens for somatic mutations on risk haplotypes. These steps are likely to be necessary to develop reliable biomarkers for therapeutics and pharmacogenetics.

In the quest to solve cryptic human complex variation, researchers in human genomics have access to a greater toolbox than ever before. Increases in sample size and meta-analyses together with a new catalogue of markers will increase power to detect rare variants. Further, efficacious whole genome sequencing and the ability to explore beyond the DNA level mean the post GWAS era promises to be one of unprecedented discovery.

## Figures and Tables

**Fig. (2):**
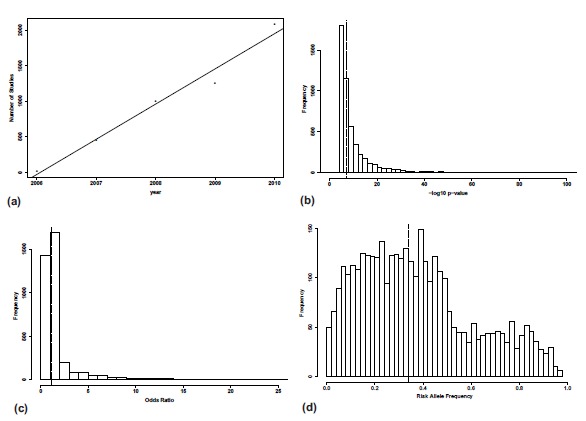
Summary statistics for entries added to NGHRi database since 2005 http://www.genome.gov/gwastudies. **a**) shows the number of studies added per year (Regression r^2^=0.97), **b**) shows the distribution of reported –log_10_ P-values – median is 7.2, **c**) shows the distribution of reported effects (e.g. odds ratio or beta) – median is 1.1, **d**) is histogram of risk allele frequencies – median is 0.34.

**Fig. (3):**
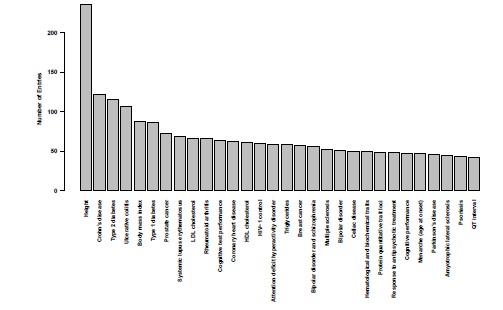
Number of entries in NHGRi database for top 30 traits from 2005-2011.

**Fig. (4):**
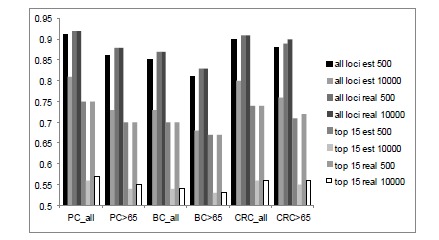
Descriminative ability (AUC) for prostate (PC), breast (BC) and colorectal cancer (CRC) for all ages and for over 65 years of age. Either 500 or 10,000 loci were simulated with estimates or real/actual values for all loci or top 15 significant results used in prediction. Prevalence used for PC, BC and CRC for (all ages, >65) were (0.007,0.04),(0.007,0.025) and (0.004,0.01) respectively and heritabilities were 0.42(PC),0.27(BC),and 0.35(CRC). Prevalences were extracted from the Information Services Division from the National Health Service Scotland (http://www.isdscotland.org/index.asp).

**Table 1.** Examples of Number of Discovered Loci and Percentage of Heritability (h^2^) Explained [22]

Disease/Trait	Number of discovered loci	% of h^2^ explained	h^2^
Type 1 diabetes	41	60	0.6
Fetal Heamaglobin levels	3	50	0.6
Macular degeneration	3	50	0.7
Type 2 diabetes	39	20-25	0.38
Crohn’s disease	71	20-25	0.5
LDL and HDL levels	95	20-25	0.3
Height	180	12	0.8

* Narrow sense heritability of trait from published literature [22].
